# Correction: Tuberculosis preventive treatment should be considered for all household contacts of pulmonary tuberculosis patients in India

**DOI:** 10.1371/journal.pone.0244914

**Published:** 2020-12-31

**Authors:** The PLOS ONE Staff

## Notice of republication

This article was republished on August 28, 2020, to correct an error in the greater-than or equal to, and less than or equal to symbols. The publisher apologizes for the error. Please download this article again to view the correct version. The originally published, uncorrected article and the republished, corrected article are provided here for reference.

## Supporting information

S1 File Originally published, uncorrected article. (PDF)

S2 File Republished corrected article. (PDF)
