# Supplementary material for: Tuberculosis preventive treatment should be considered for all household contacts of pulmonary tuberculosis patients in India
Source: PLoS One. 2020 Jul 29;15(7):e0236743. doi: 10.1371/journal.pone.0236743 (PMC7390377; doi:10.1371/journal.pone.0236743)
Supplement: S1 Table — This table shows the incidence of TB disease (iTBD) among the household contacts (HHC) of adult pulmonary TB (PTB) patients in India, stratified using different TST (≥ 5 mm, ≥ 10 mm) and/or IGRA (≥ 0.35 IU/ml, ≥ 0.7 IU/ml) cut offs to define the baseline TB infection (TBI) status. The iTBD rates were similar irrespective of the individual test cut off used to define baseline TBI, and irrespective of whether the definition used both a positive TST and IGRA test (“AND”) or either test alone (“OR”). (DOCX) [file pone.0236743.s001.docx]

| **S1 Table: Incidence rates for TB Disease Among Household Contacts of Adult Pulmonary TB Patients in India** | | | | | | |
| --- | --- | --- | --- | --- | --- | --- |
| **Definitions of Baseline TB Infection** | **N** | **iTBD n** | **Person time (years)** | **IR/1000 PY** | **Lower CI** | **Upper CI** |
| All HHC | 997 | 20 | 1602 | 12 | 8 | 19 |
| TST >5mm and/or IGRA >0.35 IU/ml | 707 | 15 | 1127 | 13 | 8 | 22 |
| TST >5mm and/or IGRA >0.7 IU/ml | 685 | 15 | 1095 | 14 | 8 | 23 |
| TST >10mm and/or IGRA >0.35 IU/ml | 553 | 11 | 882 | 12 | 6 | 22 |
| TST >10mm and/or IGRA >0.7 IU/ml | 525 | 11 | 839 | 13 | 7 | 23 |
|  |  |  |  |  |  |  |
| IGRA >0.35IU/ml irrespective of TST | 484 | 11 | 770 | 14 | 7 | 26 |
| IGRA >0.7 IU/ml irrespective of TST | 443 | 11 | 708 | 16 | 8 | 28 |
|  | | | | | | |
| TST >5mm irrespective of IGRA | 524 | 10 | 839 | 12 | 6 | 22 |
| TST >10mm irrespective of IGRA | 252 | 5 | 388 | 13 | 4 | 30 |
