# Supplementary material for: Tuberculosis preventive treatment should be considered for all household contacts of pulmonary tuberculosis patients in India
Source: PLoS One. 2020 Jul 29;15(7):e0236743. doi: 10.1371/journal.pone.0236743 (PMC7390377; doi:10.1371/journal.pone.0236743)
Supplement: S2 Table — This table shows the incident TB infection (iTBI) rates among household contacts (HHC) of adult pulmonary TB (PTB) patients in India, stratified using different thresholds for TST conversion and/or IGRA conversion to define iTBI status. The rates of iTBI were higher for lower TST/IGRA conversion thresholds as compared to higher TST/IGRA conversion; and the iTBI rates were higher when the iTBI definition used either test positive (“OR”) criteria as compared to both test positive (“AND”) criteria. In addition, changing the cut-off for a positive IGRA from ≥0.35 to ≥ 0.70 IU/ml did not significantly impact the iTBI estimates. However, increasing the induration TST cut-off from ≥ 5 mm to ≥ 10 mm or requiring a ≥ 6 mm increase in induration from previous reading resulted in lower iTBI estimates, regardless of the IGRA cut off used. (DOCX) [file pone.0236743.s002.docx]

| **S2 Table: Incidence rates for TB Infection Among Household Contacts of Adult Pulmonary TB Patients in India** | | | | | | |
| --- | --- | --- | --- | --- | --- | --- |
| **Definitions of Incident TB Infection**  **(Follow-up TST and/or IGRA results)** | **N** | **iTBI n** | **Person time (years)** | **IR/1000 PY** | **Lower CI** | **Upper CI** |
| "OR" Definitions |  |  |  |  |  |  |
| TST >5mm OR IGRA >0.35 IU/ml; | 221 | 123 | 249 | 491 | 408 | 586 |
| TST >5mm OR IGRA >0.70 IU/ml | 221 | 115 | 257 | 445 | 368 | 534 |
| TST >6mm increase OR IGRA >0.35 IU/ml | 221 | 82 | 312 | 263 | 209 | 327 |
| TST >6mm increase OR IGRA >0.70 IU/ml | 221 | 78 | 302 | 247 | 195 | 308 |
| TST >10mm OR IGRA >0.35 IU/ml | 221 | 88 | 300 | 294 | 236 | 362 |
| TST >10mm OR IGRA >0.70 IU/ml | 221 | 76 | 302 | 244 | 192 | 305 |
| "AND" Definitions |  |  |  |  |  |  |
| TST >5mm AND IGRA >0.35 IU/ml; | 183 | 13 | 316 | 41 | 22 | 70 |
| TST >5mm AND IGRA >0.70 IU/ml | 183 | 10 | 334 | 28 | 13 | 54 |
| TST >6mm increase AND IGRA >0.35 IU/ml | 183 | 6 | 307 | 19 | 7 | 41 |
| TST >6mm increase AND IGRA >0.70 IU/ml | 183 | 5 | 322 | 16 | 5 | 36 |
| TST >10mm AND IGRA >0.35 IU/ml | 183 | 6 | 307 | 19 | 7 | 41 |
| TST >10mm AND IGRA >0.70 IU/ml | 183 | 5 | 322 | 16 | 5 | 36 |
| “Only Definitions |  |  |  |  |  |  |
| TST >5mm | 209 | 100 | 267 | 375 | 305 | 456 |
| TST > 6mm increase | 209 | 59 | 325 | 181 | 138 | 234 |
| TST >10mm | 209 | 55 | 327 | 168 | 127 | 219 |
| IGRA >0.35 IU/ml | 211 | 47 | 325 | 145 | 106 | 193 |
| IGRA >0.70 IU/ml | 211 | 33 | 342 | 97 | 67 | 136 |
