# Supplementary material for: Tuberculosis preventive treatment should be considered for all household contacts of pulmonary tuberculosis patients in India
Source: PLoS One. 2020 Jul 29;15(7):e0236743. doi: 10.1371/journal.pone.0236743 (PMC7390377; doi:10.1371/journal.pone.0236743)
Supplement: S3 Table — This table shows the incident TB disease (iTBD) rates among household contacts (HHC) of adult pulmonary TB (PTB) patients in India, stratified by incident TB infection (iTBI) status which was defined using different TST conversion cut offs (≥ 5 mm, ≥ 10 mm, and ≥ 6 mm increase in induration from previous reading) along with the IGRA conversion cut off of ≥ 0.35 IU/l. The comparison shows that the iTBD estimates were similar for those HHC with and without iTBI, irrespective of the TST or IGRA cutoffs used to define iTBI. In addition, the iTBD estimates were similar irrespective of whether the definition of iTBI was based on the requirement of both a positive TST and IGRA test (“AND”) or either test alone (“OR”). (DOCX) [file pone.0236743.s003.docx]

| **S3 Table: Incident TB Disease Rates Among Household Contacts with and Without Incident TB Infection** | | | | | |
| --- | --- | --- | --- | --- | --- |
| **Definitions of Incident TB Infection**  **(Follow-up TST and/or IGRA results)** | **N** | **iTBD n** | **IR/1000 PY** | **Lower CI** | **Upper CI** |
| TST ≥5mm or IGRA≥0.35 IU/ml | 123 | 4 | 18 | 5 | 46 |
| TST <5mm or IGRA<0.35 IU/ml | 98 | 1 | 6 | 1 | 33 |
| TST ≥5mm and IGRA≥0.35 IU/ml | 13 | 1 | 42 | 1 | 236 |
| TST <5mm and IGRA<0.35 IU/ml | 170 | 2 | 7 | 1 | 23 |
|  |  |  |  |  |  |
| TST ≥6mm increase or IGRA≥0.35 IU/ml | 82 | 2 | 13 | 2 | 47 |
| TST <6mm increase or IGRA<0.35 IU/ml | 139 | 3 | 12 | 3 | 36 |
| TST ≥6mm increase and IGRA≥0.35 IU/ml | 6 | 1 | 101 | 26 | 565 |
| TST <6mm increase and IGRA<0.35 IU/ml | 177 | 2 | 6 | 1 | 22 |
|  |  |  |  |  |  |
| TST ≥10mm or IGRA≥0.35 IU/ml | 88 | 2 | 12 | 1 | 45 |
| TST <10mm or IGRA<0.35 IU/ml | 133 | 3 | 13 | 3 | 38 |
| TST ≥10mm and IGRA≥0.35 IU/ml | 6 | 1 | 101 | 26 | 565 |
| TST <10mm and IGRA<0.35 IU/ml | 177 | 2 | 6 | 1 | 22 |
